# Patient-specific fluid simulation of transcatheter mitral valve replacement in mitral annulus calcification

**DOI:** 10.3389/fcvm.2022.934305

**Published:** 2022-12-15

**Authors:** Samuel Joseph Hill, Alistair Young, Bernard Prendergast, Simon Redwood, Ronak Rajani, Adelaide De Vecchi

**Affiliations:** ^1^School of Biomedical Engineering and Imaging Sciences, King’s College London, London, United Kingdom; ^2^Cardiovascular Directorate, Guy’s and St Thomas’ NHS Foundation Trust, London, United Kingdom

**Keywords:** TMVR in MAC, computational simulation, subvalvular thrombosis, LVOTO, blood residence time, wall shear stress

## Abstract

**Introduction:**

Transcatheter mitral valve replacement is a promising alternative to open-heart surgery in elderly patients. Patients with severe mitral annulus calcification (MAC) are a particularly high-risk population, where postprocedural complications can have catastrophic effects. Amongst these, obstruction of the left ventricular outflow tract can lead to ventricular hypertrophic remodeling and subsequent heart failure, while subclinical valve thrombosis can result in early bioprosthetic valve failure.

**Methods:**

To elucidate the mechanisms of left ventricular outflow tract obstruction and valve thrombosis following valve-in-MAC procedures, we used image processing and Computational Fluid Dynamics (CFD) software to generate patient- and device-specific models based on preprocedural CT data. Personalized computer simulations were performed to predict the left ventricular haemodynamics after implantation in three patients with severe MAC.

**Results:**

The simulations have successfully captured the increased pressure gradient in the left ventricular outflow tract as a result of the partial obstruction due to the implanted valve. Regions of wall shear stress above the threshold value for platelet activation were also observed on the bioprosthetic frame as a result of the reduced outflow tract area, which led to increases in flow resistance and blood residence time inside the ventricle. Consistent with these findings, areas of slow recirculating flow and blood stasis formed near the valve frame, creating potential pro-thrombotic conditions.

**Discussion:**

This study provides insight into the relationship between size and shape of the outflow tract post-implantation, pressure gradients and pro-thrombotic flow metrics such as wall shear stress and blood residence time. Results show the potential of CFD modeling to bring key functional metrics into preprocedural assessment for a comprehensive evaluation of post-procedural risks beyond anatomical factors. Following further validation and extension to the atrial chamber, this approach can provide an in-depth analysis of the likelihood of valvular thrombosis.

## Introduction

Mitral annulus calcification (MAC) is the deposition of calcified material around the mitral annulus leading to a reduced orifice area and stiffening of the surrounding tissue, which can lead to concomitant valvular involvement (1). This chronic disease is found in 10% of patients over the age 60 and 33% of those over 90 years (2), thus patients are often at very high risk for surgery. In recent years, there has accordingly been interest in the transcatheter deployment of valves inside the MAC to treat significant mitral regurgitation or stenosis (3, 4). Despite the promise of this non-surgical technique, this procedure is still associated with a 1-year outcome of all-cause mortality reported as 34.5% (5). The increased morbidity and mortality associated with transcatheter techniques is likely associated with sub-optimal pre-procedural planning due to the high heterogeneity of MAC in terms of its density and distribution and its varying interplay with the mitral valve apparatus. Furthermore, transcatheter heart valves used for Valve in MAC (ViMAC) procedures are either valves designed for the aortic position or for patients with mitral valve regurgitation without significant MAC involvement (6, 7).

The main risks associated with ViMAC in the transcatheter mitral valve replacement (TMVR) Global registry (30-day outcomes) were stroke (3.9%), mitral valve re-intervention (13.8%), persistent mitral regurgitation (13.2%), valve embolism (6.9%) and LVOT obstruction (39.7%) (8). Of these left ventricular tract obstruction (LVOTO) is the most prevailing reason for which patients are declined treatment. LVOTO can lead to emergency reintervention or, if left unchecked, to ventricular maladaptation and failure (9–12). As the native leaflets are not generally resected in TMVR, the anterior leaflet can be displaced toward the LVOT by the device frame. This can result in elongation and narrowing of the LVOT, with formation of the so-called neo-LVOT, which ultimately causes dynamic LVOTO (13, 14). Assessment of LVOTO using gated multiphase computed tomography (CT) is common practice in a clinical setting (15, 16). Currently the degree of obstruction is estimated by embedding a cylindrical model of the device of choice in the preprocedural CT data to calculate the neo-LVOT area after deployment (14). A neo-LVOT area < 180–200 mm^2^ is currently considered to lead to an unacceptable degree of obstruction (17, 18). A small neo-LVOT area can also result from other patient-specific anatomical factors known to influence the risk of obstruction including the aortomitral angulation, the presence of the septal bulge, basal cavity size and the left ventricle (LV) anatomy. However, besides the current anatomy-based assessment of LVOTO risks, physiological pressure gradients are increasingly recognized as a true measure of obstruction. Post-procedural criteria indicating excessive obstruction are a pressure gradient in the LVOT greater than 50 mmHg or an increase of 10 mmHg or more from the preprocedural baseline – both evaluated based on Doppler data (16, 19). However, Doppler-derived measurements can overestimate the pressure gradient and can only be computed once the device is in place (20–22). Whilst measuring the neo-LVOT from preprocedural CT data is common practice, the threshold for LVOTO is only a crude approximation of the subsequent haemodynamic changes and is not indexed based on the patient’s other anatomical metrics. This type of analysis may thus leave relevant heamodynamic risk factors unaccounted for, potentially leading to inaccurate assessment.

Another risk associated with any TMVR procedure is early bioprosthetic valve failure. Although in part this can be related to bioprosthetic valvular thrombosis, this risk is largely mitigated by long term anticoagulation (23–26). Without long-term anticoagulation, 12.7% of explanted bioprosthesis showed evidence of thrombosis (median time 24 months) (23). Despite the clear association of valve thrombosis to early valve failure, little is known as to whether or not ventricular oscillatory flow patterns on the underside of the bioprosthetic valve leaflets result in subclinical changes and promote degenerative change. Early data suggests that the accumulation of fibrinogen, a key thrombogenic protein, on the valve structure has been observed in the case of bioprostheses (27). Regions of high wall shear stress (WSS) can increase platelet activation and initiate thrombogenesis in areas of the valve where fibrin gel has formed (25). Similarly, areas of oscillatory flow and recirculation of slow flow with high blood residence time (BRT) inside the LV (i.e., the time blood particle spend inside of the ventricle before being ejected) also provide favorable conditions for aggregation of platelets and proteins into a thrombus.

The principal aim of the current study is to evaluate whether computation fluid dynamics can provide patient specific physiological models of ViMAC and thereby predictions of anticipated left ventricular outflow tract gradients from preprocedural multiphase gated CT scans. The secondary aim was to explore whether blood flow biomarkers from the models could be used to evaluate prothrombotic conditions on the underside of bioprosthetic mitral valve leaflets (28–31).

## Materials and methods

### Patient data

Three patients diagnosed with severe MAC were selected for the study (Table 1), with ages 61, 70, and 90 (VIM-1, VIM-2, and VIM-3, respectively). VIM-1 and VIM-2 were recommended for ViMAC after exhibiting mitral regurgitation, while VIM-3 showed significant mitral stenosis. No patients had a history an ischemic heart disease and all native anatomy was suitable for ViMAC TMVR with a Sapien 3 bioprosthetic device. All patients were evaluated in a dedicated transcatheter mitral valve clinic. No patient had symptoms of unstable angina and all were appropriately revasculazized prior to any procedure. VIM-1 and VIM-2 subsequently underwent mitral valve replacement, while VIM-3 did not progress to TMVR due to concerns of severe LVOTO. The main inclusion criteria for transcatheter ViMAC were the presence of severe symptomatic valvular disease with severe MAC and the high surgical risk caused by comorbidities or technical difficulties, as well as assessed by the heart team at Guy’s and St Thomas’ NHS Trust. Exclusion criteria were a LV ejection fraction < 25%, a LV end diastolic diameter > 7.0 cm, concurrent aortic or tricuspid valve disease requiring intervention, and presence of non-cardiac co-morbidities that are likely to negatively affect the post-procedural life expectancy.

**TABLE 1:** Baseline resting patient data.

	VIM-1	VIM-2	VIM-3
Sex	Male	Female	Female
Age	70	61	90
Ejection fraction (%)	43	45	65
Neo-LVOT area (mm^2^)	268.6	521.2	209.2
MAC thickness (mm)	6.52	6.27	8.21
MAC height (mm)	5.87	2.98	7.80
Circumference of mitral valve encompassed by MAC (°)	332.59	322.89	273.56

LVOT, left ventricular outflow tract; MAC, mitral annulus calcification.

### Imaging protocols and analysis

All cardiac CT scans were performed on a Siemens SOMATOM Force dual source scanner (Siemens, Germany). A low dose non-contrast scan was used to reduce the range of retrospective scanning and to evaluate for mitral valve annulus calcification. Following this, a retrospective contrast enhanced ECG gated helical acquisition was performed in a single breath hold with bolus tracking in the ascending aorta to optimize contrast enhancement in the left atrium and over the mitral valve. Scanning parameters included a heart rate dependent pitch (0.2–0.45), gantry rotation time of 250 ms tube voltage of 100 or 120 kVp, depending on the patient’s body-mass index and a tube current of 125–300 mA. A biphasic contrast protocol was used with 90 ml of iodinated contrast at a flow rate of 5 ml/s followed by 40 ml of saline at 5 ml/s. The acquired cardiac CT data was reconstructed using a medium level of advanced model-based iterative reconstruction with the use of a 250 mm field of view, 512 × 512 matrix and a smooth reconstruction kernel. In the presence of significant ectopy, ECG-editing was performed using vendor-specific software. All ventricular measurements were taken at the peak systolic CT frame in line with previous protocols for evaluation of the LVOT obstruction (14, 16).

**FIGURE 1:**
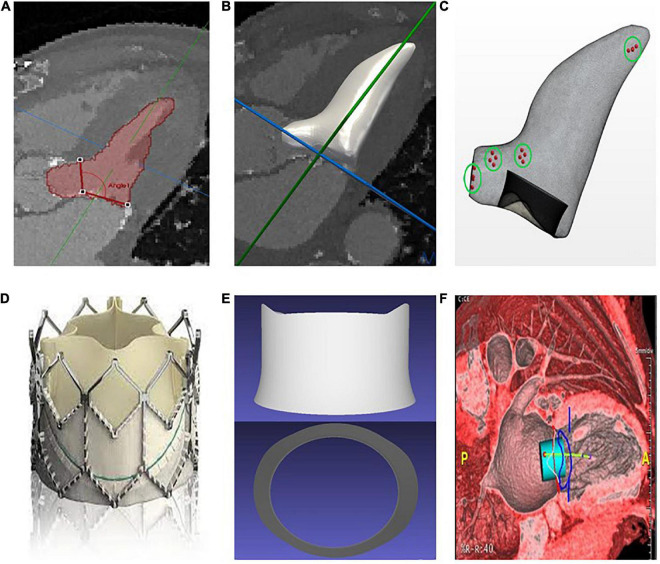
**(A)** Manual segmentation of the left ventricle and measurement of the aortomitral angle. **(B)** Interpolation and smoothing of the segmented contours to a 3D surface. **(C)** Generation of a surface mesh, from which a volume mesh is generated (simulation domain); the points in the green circles represent the areas where the pressure is measured. **(D)** Sapien 3 bioprosthesis. **(E)** Simplified Computer-Aided Design (CAD) model of the Sapien 3 bioprosthesis. **(F)** Preprocedural assessment on CT data with a cylindrical valve overlaid to the images for neo-LVOT measurement.

The blood pool of the LV was segmented with manual contouring at the peak systolic-frame in the multiphase CT series using the software MITKWorkbench (32), as shown in Figure 1A. A 3D surface mesh representing the endocardium was generated using a radial basis function algorithm to interpolate between 2D contours (Figure 1B). The surface mesh was then smoothed using a Laplacian smooth filter to average vertex positions with weighted positions of neighboring vertices and subject to isotropic explicit remeshing, increasing the surface vertex density to approximately 100,000 nodes. To verify the accuracy of the surface mesh, measurements were taken of key anatomical metrics such as the LV length and diameter and aortomitral angulation, and compared to the measurement on the imaging data (see Table 2). Probe points were placed throughout the fluid domain to measure the pressure gradients in specific locations. Measurements were taken at the mitral valve (MV), aortic valve (AV) and apical region (AP) of the ventricle, as well as 5 mm distally and proximally of the vena contracta between the bioprosthetic valve and the septal bulge on the LV wall (Figure 1C). Blood residence time (BRT) was measured by introducing a passive scalar representative of the age of flow inside the ventricle throughout the simulation, in order to identify areas of stagnation in the blood pool. WSS and OSI were also measured on the device frame to identify pro-thrombotic conditions in the flow. The exposure time to maximum WSS was quantified as the length of time where the WSS magnitude was above the threshold value for platelet activation as identified in experimental set-ups (33–38).

**TABLE 2:** Comparison of anatomical parameters between CT and surface mesh measurements.

	VIM-1		VIM-2		VIM-3	
	**CT**	**Model**	**CT**	**Model**	**CT**	**Model**
LV systolic diameter (mm)	26.1	26.92	29.1	29.29	29.4	29.32
Aortomitral angulation (°)	113.11	113.23	108.96	110.68	130.54	128.76
Septal distance (mm)	18.7	18.54	19	20.53	19.6	20.23

LV, left ventricle.

Wall motion tracking was performed using temporally sparse free-form deformation to create a vector field of displacement values, which were then applied to the surface mesh, deforming the initial mesh to match the wall motion at each time frame in the CT series (39). To validate the choice of parameters for the wall motion tracking, the accuracy of the deformation was evaluated in one representative case against manual segmentation of each CT frame. The corresponding Hausdorff distance was then calculated, resulting in an average value of 1.78 ± 0.76 mm over the cardiac cycle. The bioprosthetic device used was represented by a Computer-Aided Design (CAD) model of the frame of a Sapien 3 device, with height and radius matching those specified by the manufacturer for the model under consideration for each patient (Figures 1D, E). The device was then overlaid to the CT data to measure the neo-LVOT area using the Aquarius software (TeraRecon Inc., Durham, USA), as shown in Figure 1F.

### Patient-specific flow modeling

From the end-systolic surface mesh a polyhedral volume mesh was created using the commercial software STAR-CCM+ (Siemens PLM). A CAD model of the Sapien 3 was embedded into the fluid domain in the position of the mitral annulus prior to volumetric meshing (Figure 1C). Computational Fluid Dynamics (CFD) simulations based on the patient-specific anatomy and boundary conditions were performed using STAR-CCM+, solving for the incompressible Navier-Stokes equations with blood density 1,060 kg/*m*^3^ and viscosity 3e−3 Pa.s.

A mesh independence study was performed to assess the optimal size of the mesh. The final volumetric mesh for the LV blood pool comprised an average of 1,100,000 elements, depending on the size of the LV, with a base size in the range of 0.5 mm. Volumetric growth rate was 1.1, constricting the increase in element size toward the centre of the ventricle.

Boundary conditions were derived from the preprocedural CT datasets. Specifically, the mass flow rate of blood through each of the mitral and aortic valves was derived from the change in mesh volume over each time step, and the deformation field extracted from the wall motion tracking was prescribed on the endocardial wall.

Aortic jet peak velocity and the peak pressure gradient in the LVOT were measured from the simulation data and compared to post-procedural Doppler derived values for validation (Table 3).

**TABLE 3:** VIM-1 model validation against Doppler-derived values.

	Echo-Doppler	Simulation	Error
Peak velocity at aortic valve (m/s)	1.6	1.62	1.25%
Peak pressure gradient in LVOT (mmHg)	10.40	9.96	4.23%

## Results

**FIGURE 2:**
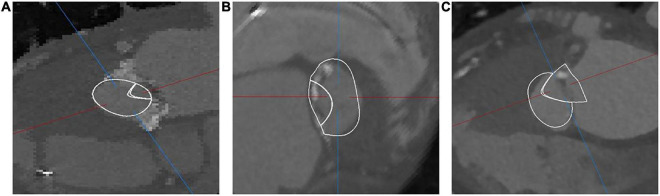
Overlay of computational domain onto CT depicting the shape of the neo-LVOT in VIM-1 (A), VIM-2 (B), and VIM-3 (C).

Pressure gradients were measured over the systolic period in four locations, with mean and peak values reported in Table 4 along with the WSS values on the device. The maximum LVOT pressure gradient was below the threshold value for LVOT obstruction (50 mmHg) for cases VIM-1 and VIM-2 but above it for VIM-3, where it reached 80 mmHg. Proportionality was observed between the pressure gradients in the LVOT and the magnitude of maximum WSS in all cases, whereas an inverse relationship was found between the magnitude of the pressure gradients and the area of the neo-LVOT. The shape of the neo-LVOT after implantation was extracted for each patient from the corresponding computational models at peak systole, in a cross-section perpendicular to the neo-LVOT axis (Figures 2A–C).

**TABLE 4:** Pressure gradients and wall shear stress data.

	VIM-1	VIM-2	VIM-3
Max systolic PG LVOT (mmHg)	19.5	2.5	80.6
Mean systolic PG LVOT (mmHg)	14.6	2.0	45.2
Max WSS (Pa)	98.2	38.8	169.8
Exposure time (s)	0.16	0.26	0.03
Mean WSS (Pa)	52.0	24.6	77.6
Max surface averaged WSS (Pa)	9.8	3.6	13.5

PG, pressure gradients; LVOT, left ventricular outflow tract; WSS, wall shear stress.

**FIGURE 3:**
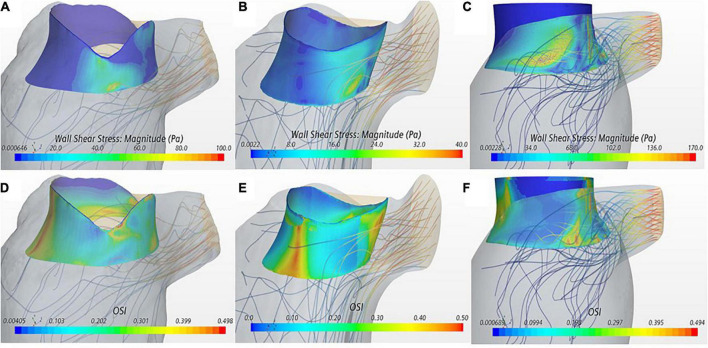
(A–C) Wall shear stress (WSS) displayed on the device frame for cases VIM-1:3 (left to right). (D–F) OSI displayed on the device frame for cases VIM-1:3 (left to right). Streamlines from the aortic valve with a backward integration are included in each, velocity of the streamline is represented from low (blue) to high (red).

Wall shear stress magnitude was visualized on the device frame as a surrogate marker for platelet activation (Figures 3A–C). All three cases showed a region of sharp increase in WSS values on the valve frame near the LVOT in the wake of the aortic outflow jet. VIM-3 showed a WSS value that was 72 and 337% higher than VIM-1 and VIM-2, respectively, as well as the presence of vortical structures in the LVOT.

The OSI quantifies the oscillatory nature of the flow with low values indicating predominantly unidirectional flow. OSI iso-contours were used in conjunction with streamline analysis to identify areas of blood recirculation on the device frame (Figures 3D–F). All cases showed areas of low OSI (i.e., <0.1) that coincided with the region of high WSS magnitude near the LVOT. In contrast, the septal side of the device frame, opposite to the LVOT, exhibited low WSS and OSI close to the maximum value of 0.5, signaling oscillatory flow; streamlines in these regions confirmed presence of recirculation flow. In all cases the maximum WSS and minimum OSI occurred during peak ejection when the aortic jet was at maximum velocity.

**FIGURE 4:**
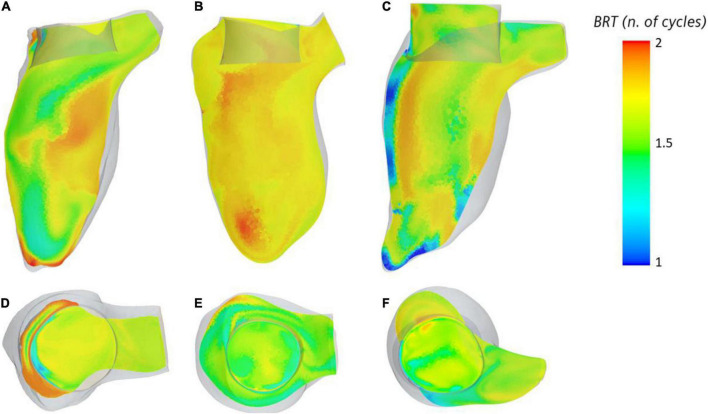
(A–C) Blood residence displayed inside the ventricle on the longitudinal axis for cases VIM1-3 (left to right), (D–F) blood residence time inside and around the device frame for cases VIM-1:3 (left to right).

The BRT throughout two cardiac cycles was measured in the ventricular cavity (Figures 4A–C) to identify areas of stagnation in the blood pool near the valve. Within the device frame, the mean BRT was 1.51 s for VIM-1 (1.5 ± 0.04 cardiac cycles), 1.65 for VIM-2 (1.51 ± 0.04 cardiac cycles) and 0.86 s for VIM-3 (1.3 ± 0.25 cardiac cycles). VIM-1 shows localized areas of high BRT (1.87 cardiac cycles) coinciding with low flow velocities at the apex and between the device frame and anterior wall and outside the device frame adjacent to the apical wall (Figures 4A, D). VIM-3 also showed localized regions inside the device frame where blood resided for 1.8–2 cardiac cycles (Figures 4C, F), in contrast to VIM-2, where the maximum BRT near the valve was significantly lower (1.6 cardiac cycles, Figures 4B, E).

**FIGURE 5:**
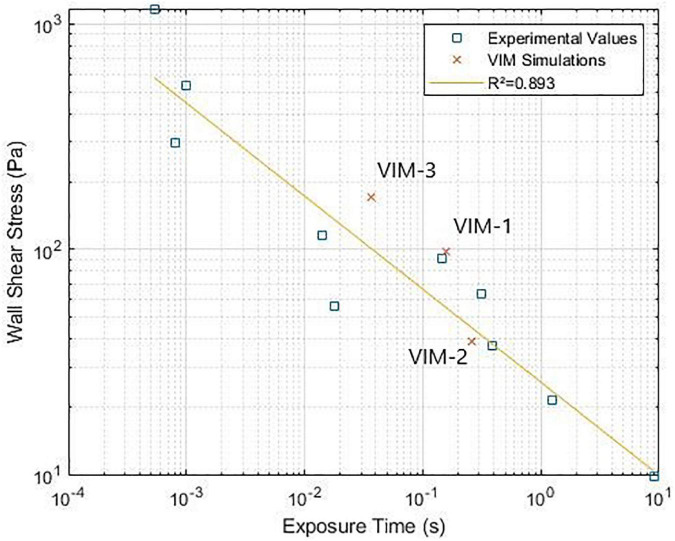
(Wall shear stress-exposure time threshold for platelet activation. Comparison between experimental data from literature and simulation results (33–38).

Finally, Figure 5 reports the values of maximum WSS on the valve frame and the corresponding exposure time. The results for VIM-1, VIM-2, and VIM-3 (orange dots) were compared to experimental values of WSS and exposure time that resulted in platelet activation in blood from literature (33–38).

## Discussion

The principal findings of the current study are that a relationship exists between LVOT pressure gradient, size and shape of the neo-LVOT area and pro-thrombotic flow metrics such as WSS and OSI. Further, the size and shape of a bioprosthetic in relation to the ventricle wall impact ejection dynamics and BRT, and the WSS can reach values close to the threshold for platelet activation on the device frame during ejection.

In line with previous findings from both clinical and computational studies, LVOT pressure gradient was inversely proportional to neo-LVOT area for all cases (10, 15, 40). There was also a direct proportionality between the magnitude of WSS on the device and the pressure gradient in the LVOT. These findings are consistent with the reduced cross-sectional area in the LVOT, increasing blood flow velocity and hence shear stresses on the device frame. Although VIM-1 and VIM-3 exhibit a similarly sized Neo-LVOT, results of WSS, PG and BRT varied significantly. The maximum WSS and PG in the LVOT were 1.7 and 4 times higher in VIM-3 than in VIM-1. A possible explanation for these discrepancies lies in the shape of the neo-LVOT, which exhibits a regular shape in VIM-1 with minimal protrusion of the device frame into the neo-LVOT (Figure 3A) compared to the shape in VIM-3, where the device protrudes more and is closer to the side wall (Figure 3C). This asymmetric area reduction can lead to localized increases in flow velocity and consequently WSS on the device as the flow enters the LVOT through the narrow gap on the side of the bioprosthesis. The corresponding flow dynamics are more disorganized in VIM-3 with formation and breakdown of smaller vortical structures in the LVOT, which lead to energy loss due to friction and convective effects that increase the pressure gradient for ejection. Further, BRT was increased inside the device frame in VIM-3 as opposed to between the device and apical wall in VIM-1. In this latter case, the gap between the device frame and the posterior and lateral wall resulted in a pocket of stagnating blood with low velocity and high BRT. These results suggest that in these three cases, not only the size of the neo-LVOT but also its shape and how the device sits in the annulus with respect to the surrounding ventricle wall should be assessed to personalize preprocedural assessment for TMVR. This is an exciting hypothesis that, if validated on a larger patient cohort, could improve existing preprocedural planning based on anatomy scans.

Finally, in all of our models the combination of WSS magnitude and exposure times on the valve was found to be close to the threshold values for platelet activation measured in experimental settings (shown in Figure 5), suggesting that there is potential for thrombus formation on the device in these patients. In our cases, the regions of high WSS occurred in proximity to regions with OSI values close to 0.5, which indicated oscillating flow. Streamline analysis in these areas showed the presence of recirculating regions inside the valve frame, suggesting that platelets could be activated by the high shear conditions and then slowed down inside the bioprosthetic frame where aggregation and initiation of thrombus formation can occur (38). Contact with a foreign body can also trigger thrombogenesis, although this mechanism is attenuated, albeit not absent, in bioprostheses compared to mechanical devices. It is also worth noticing, however, that regions of high WSS tend to occur near in the neo-LVOT and involve flow that is being ejected, meaning that shear-activated platelets are likely to leave the ventricle soon after being subjected to high WSS. This is not the case for the blood exposed to high WSS on the inner side of the device and then trapped inside the valve frame without being ejected. In particular the fact that the native anterior leaflet is not resected and adheres to the valve frame, may result in flow directed toward the aortic valve being deflected inside the bioprosthesis. This hypothesis is corroborated by the fact that the age of the flow inside the cylindrical structure of the valve tends to be higher than in that of blood outside of the device. This was specifically noted in the case of VIM-3, which exhibited the highest maximum values of WSS and also showed a localized area of increased BRT (1.8 cardiac cycles, as opposed to approximately 1.5 in VIM-1 and VIM-2), suggesting that previously activated platelets could stagnate in this region. VIM-3 also exhibited the smallest neo-LVOT area, the largest aortomitral angle and the smallest aortic valve of the three cases. The combination of these factors could have contributed to the observed results. Further studies are necessary to assess whether these conditions are sufficient to initiate valvular thrombosis.

This type of personalized modeling provides insight into blood flow metrics whose measurement is challenging to obtain in a clinical setting without the use of specialized sequences, currently applied in research settings only (e.g., Phase Contrast MRI). Such approach has shown merit in displaying the potential of LVOT obstruction to exacerbate risks of thrombus formation on the device, as well as in informing which patients may require preprocedural septal modification prior to ViMAC procedures, such as with alcohol septal ablation or leaflet modification procedures with the LAMPOON technique.

### Limitations

The CAD model of the bioprosthetic device is simplified and does not account for moving leaflets and details in the different frame components. Consideration was not made to the layers of device design including the cobalt-chromium frame, trileaflet bovine pericardial tissue valve, and polyethylene terephthalate (PET) fabric skirt, in which small particles may become trapped. A more realistic device model could therefore increase the amount of blood trapped by the different elements composing the device and hence our simplified approach could represent a “best case scenario.” Papillary muscles are excluded from the ventricle segmentation and the endocardium surface is smoothed to reduce likelihood of numerical instability. However, these approximations should result in an attenuation of the flow disturbances caused by the valve implantation, also suggesting that our value could underestimate the magnitude of pressure gradients and WSS, as well as the potential for blood flow to adhere to the valve structure. The accuracy of the ventricle motion is limited by the number of CT time-frames obtained at the time of capture, which is 10 frames for each case. Segmented surfaces are subject to interpolation between CT frames to a temporal resolution of 0.5 ms, therefore there may be loss of accuracy in the deformation field prescribed on the surface of the model (endocardium) and subsequently in the flow simulation. Finally, the sample size of the study is limited to three patients due to the availability of ViMAC patient data, not allowing the comparison between patients with significant LVOTO and those without.

## Conclusion

The methodology presented has successfully captured the increased pressure gradient and maximum WSS as a result of reduced neo-LVOT area, and also provided insight into the mechanistic interpretation of OSI, WSS and BRT on the underside of the valve leaflets that is increasingly observed in transcatheter bioprosthetic valves. This shows the potential of CFD analysis, once validated on a larger patient cohort, to complement image-based preprocedural assessment by providing important information on the patient-specific haemodynamics, as well as to shed light on mechanisms of potential thrombus formation on the valve.

## Data availability statement

The original contributions presented in this study are included in this article/supplementary material, further inquiries can be directed to the corresponding author.

## Ethics statement

Ethical review and approval was not required for the study on human participants in accordance with the local legislation and institutional requirements. Written informed consent for participation was not required for this study in accordance with the national legislation and the institutional requirements.

## Author contributions

SH, AD, and RR designed and wrote the study. SH and AD performed and analyzed the simulations. AY, BP, and SR contributed to the reviewing and editing of the manuscript. All authors contributed to the article and approved the submitted version.
