# Erratum: Patient-specific fluid simulation of transcatheter mitral valve replacement in mitral annulus calcification

**DOI:** 10.3389/fcvm.2023.1190028

**Published:** 2023-04-03

**Authors:** Frontiers Production Office

**Affiliations:** Frontiers Media SA, Lausanne, Switzerland

**Keywords:** computational simulation, subvalvular thrombosis, LVOTO, blood residence time, wall shear stress

An Erratum on Patient-specific fluid simulation of transcatheter mitral valve replacement in mitral annulus calcification By Hill SJ, Young A, Prendergast B, Redwood S, Rajani R and De Vecchi A. (2022) Front. Cardiovasc. Med. 9:934305. doi: 10.3389/fcvm.2022.934305

Due to a production error, the following funding acknowledgment was missed from the article: “Funded by the EPSRC Research Council, part of the EPSRC DTP, grant Ref: [EP/R513064/1].” The publisher apologizes for this mistake.

The original version of this article has been updated.

